# Supplementary figures and images for: Inducible CRISPR activation screen for interferon-stimulated genes identifies OAS1 as a SARS-CoV-2 restriction factor
Source: PLoS Pathog. 2022 Apr 14;18(4):e1010464. doi: 10.1371/journal.ppat.1010464 (PMC9041830; doi:10.1371/journal.ppat.1010464)

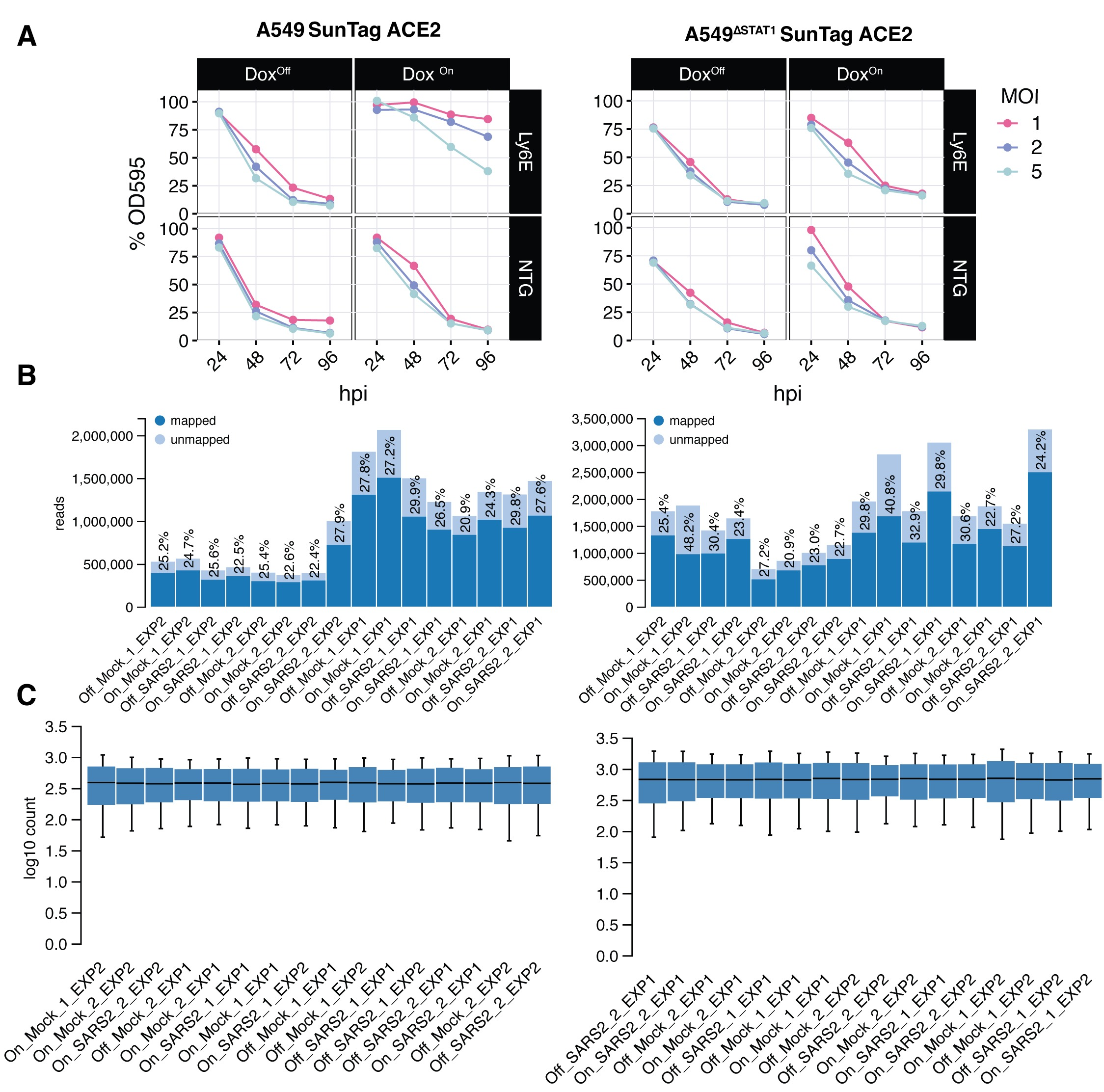

Supplement: S1 Fig — (A) Pilot experiment using Methylene Blue assay to assess SARS-CoV-2 CPE under different infection conditions. A549-SunTag ACE2 and A549ΔSTAT1-SunTag ACE2 cells, expressing LY6E gRNA or a non-targeting gRNA (NTG) were infected with SARS-CoV-2 at indicated M.O.I., fixed at indicated time points, and stained with methylene blue. Values indicate percent OD595 absorption relative to time point = 0 (set to 100%). (B) CRISPRa screen quality metrics: sequencing reads per sample. Values indicate number of reads sequenced for each sample in the pooled screens. Percentage values (light fill) for reads that fail to map to gRNA sequences in the ISG library reference. (C) CRISPRa screen quality metrics: Normalized read count distribution per sample. Log10 transformed read count for each sample normalized to the count of non-targeting guides. (TIF) [file ppat.1010464.s001.tif]

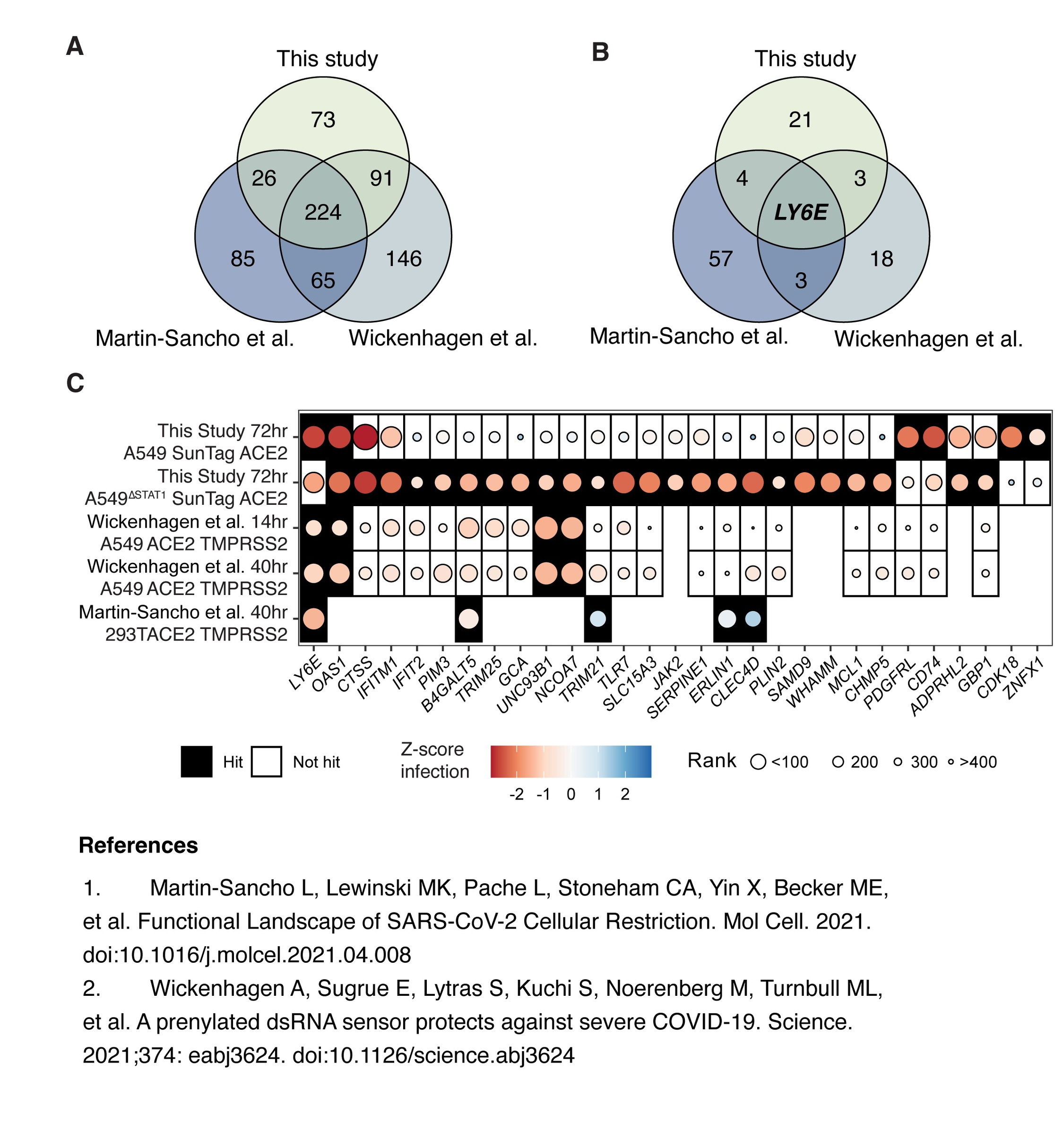

Supplement: S2 Fig — (A) Venn diagram generated from intersecting lists of ISGs from the libraries of Wickenhagen et al. [31], Martin-Sancho et al. [29] and our ISG library. (B) Similar to A, highlighting shared and distinct antiviral hits. (C) Comparison of candidate antiviral hits from published studies and this study. Z scores of infections were calculated from published datasets and ranked. Black square: antiviral hit. White square: not antiviral hit. Empty square: not tested/no data available. (TIF) [file ppat.1010464.s002.tif]

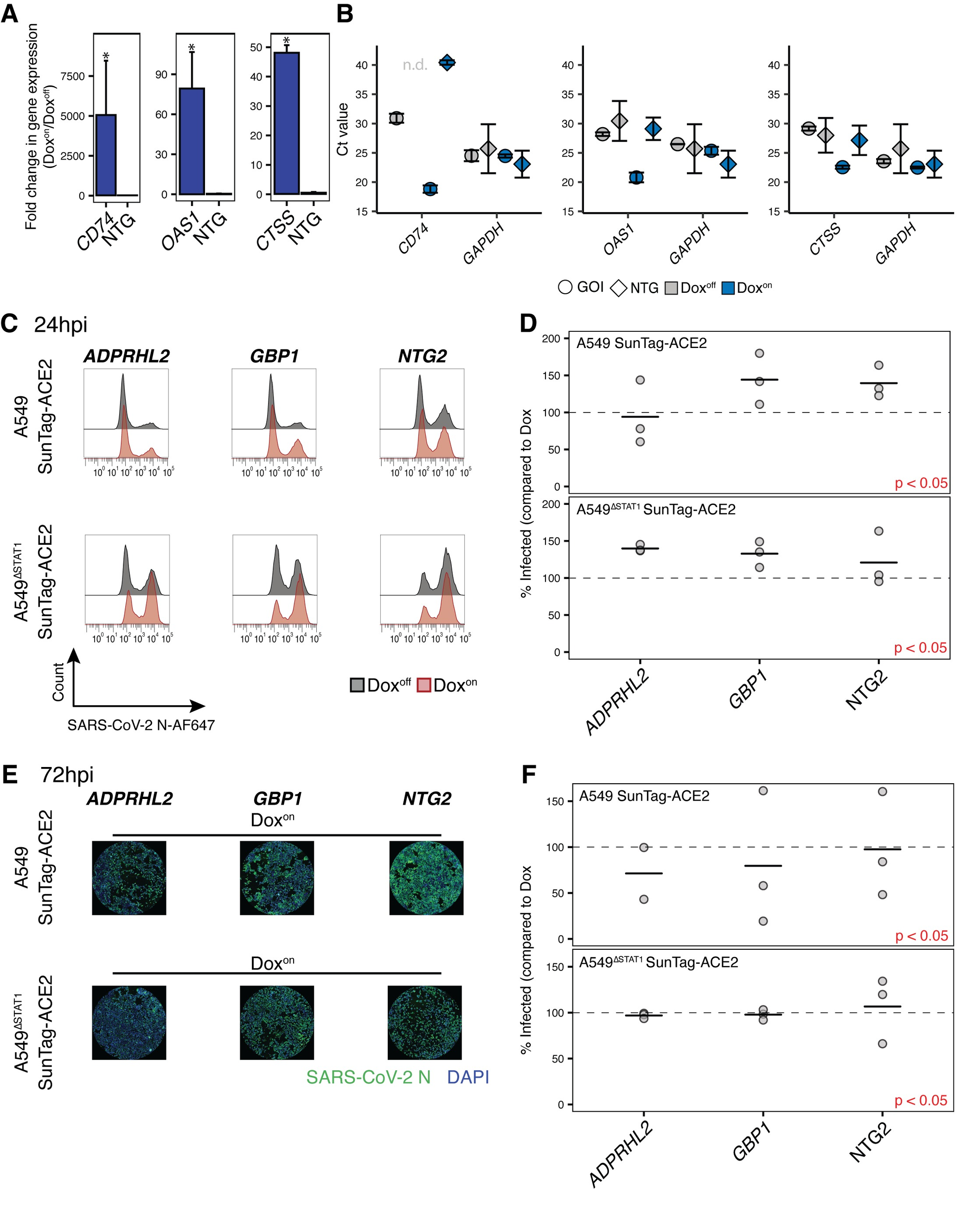

Supplement: S3 Fig — (A) qRT-PCR analysis demonstrates effective CRISPRa gene induction in A549-SunTag ACE2 cells. Values indicate fold change expression (Doxon relative to Doxoff) for indicated genes (CD74, OAS1 and CTSS) in A549-SunTag ACE2 cells expressing either corresponding gRNA or NTG. Fold change values calculated using the ΔΔCt method with GAPDH as normalization control. Undetectable Ct value for Doxoff condition of cells expressing NTG and probed for CD74 expression was set to 40 to enable fold change calculation. (B) qRT-PCR mean threshold cycle (Ct) values for CD74, OAS1, CTSS and GAPDH in A549-SunTag ACE2 cells expressing gRNA against gene of interest (GOI, circle) or NTG (diamond) in Doxon and Doxoff cells. Error bars indicate ± SD Ct value. (C) Representative flow cytometry histograms for SARS-CoV-2 N protein in A549-SunTag ACE2 and A549ΔSTAT1-SunTag ACE2 transduced with indicated gRNAs, treated (red) or not treated (gray) with Dox, at 24 hours post-infection with SARS-CoV-2 (M.O.I. = 2). (D) Percent of infected (SARS-CoV-2 N protein positive) cells quantified across biological replicates (n = 3) for experiments described in (C). Values denote percent of infected cells in Doxon cultures relative to paired Doxoff cultures. Points represent individual biological replicates, black lines indicate mean values of biological replicates for each indicated ISG gRNA. Red points indicate statistical significance (p < 0.05) as determined by paired ratio Student’s t-test. (E) Representative immunofluorescence images for SARS-CoV-2 N protein and DAPI in A549-SunTag ACE2 and A549ΔSTAT1-SunTag ACE2 transduced with indicated gRNAs and treated with Dox, at 72 hours post-infection with SARS-CoV-2 (M.O.I. = 2). (F) Percent of infected (SARS-CoV-2 N protein positive) cells quantified across biological replicates for experiments described in (E). Values denote percent of infected cells in Doxon cultures relative to paired Doxoff cultures. Points represent individual biological replicates, blac [file ppat.1010464.s003.tif]

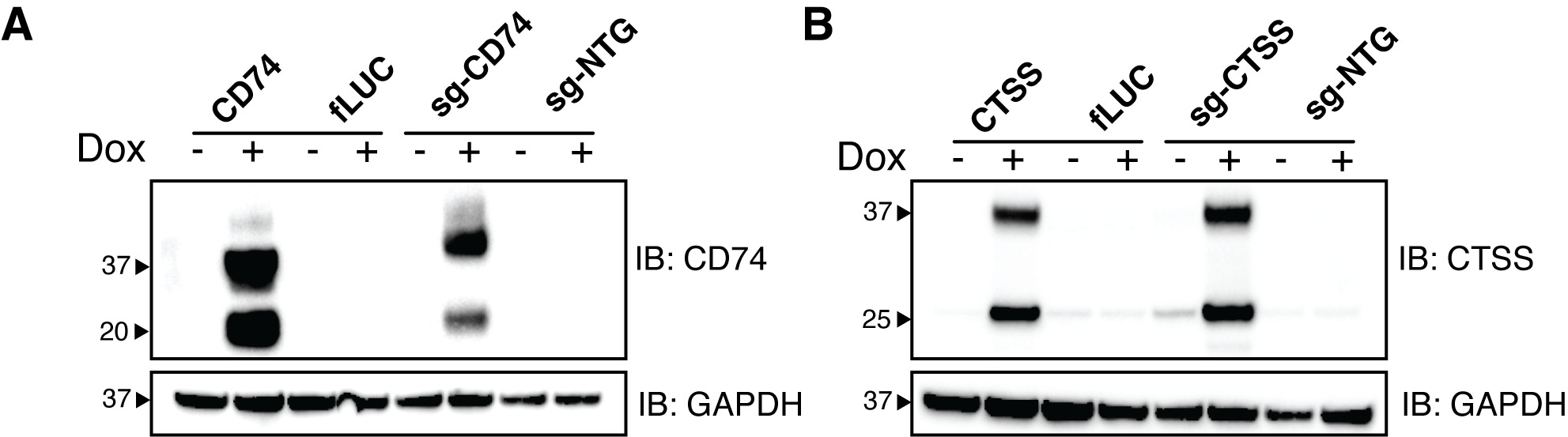

Supplement: S4 Fig — (A-B) Immunoblot analysis of CD74 (A) and Cathepsin S (B) expression in A549-ACE2 cells transduced with lentivirus encoding indicated ORFs compared to A549-SunTag ACE2 cells expressing guides targeting the activation of CD74, CTSS or a non-targeting guide. Cells were incubated with Dox to induce gene expression for 48 hours prior to processing for immunoblot analysis with the indicated antibodies. (TIF) [file ppat.1010464.s004.tif]

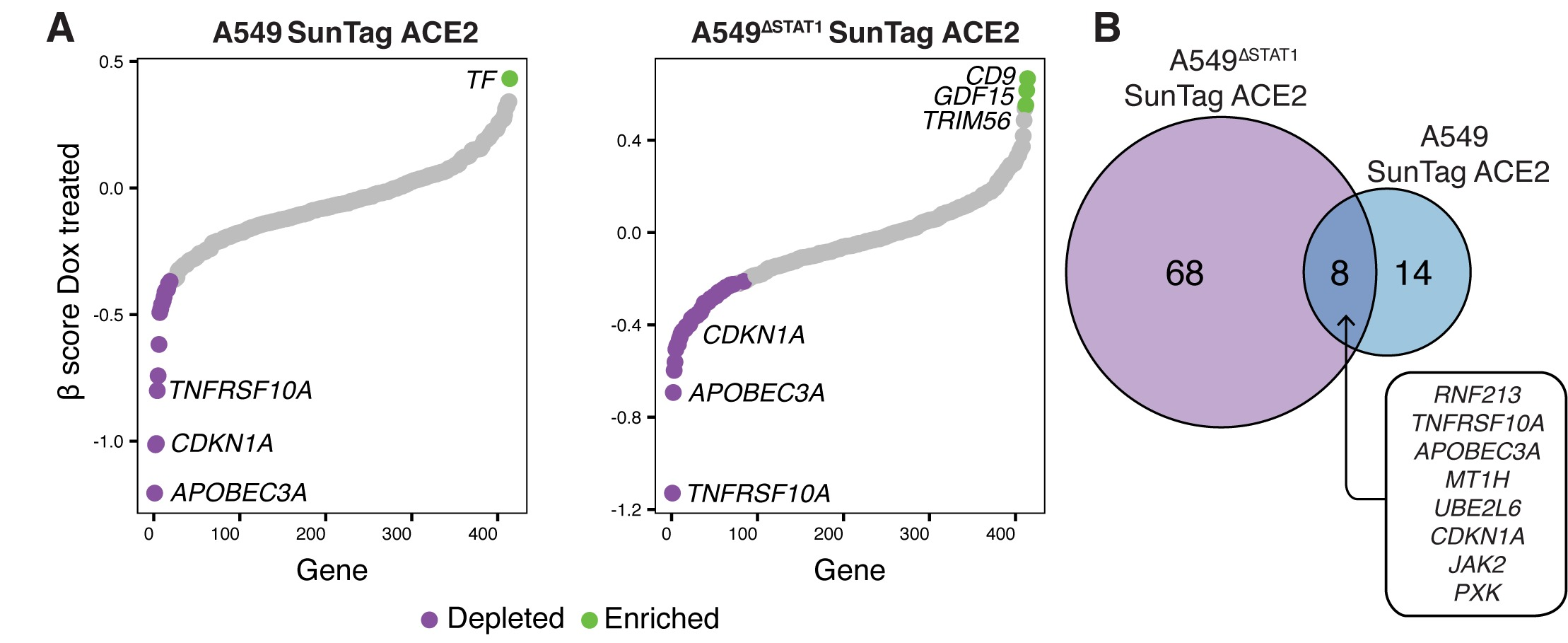

Supplement: S5 Fig — (A) ISGs ranked by the inverse of their β-scores for the Dox status coefficient (DoxOff mock infected vs DoxOn mock infected). Significantly (adjusted p value < 0.1) enriched/depleted gRNAs are highlighted in green/purple respectively. (B) Venn diagram of significantly depleted (i.e. candidate antiproliferative/proapoptotic) ISG hits from (A) in A549-SunTag ACE2 and A549ΔSTAT1-SunTag ACE2 screens. (TIF) [file ppat.1010464.s005.tif]

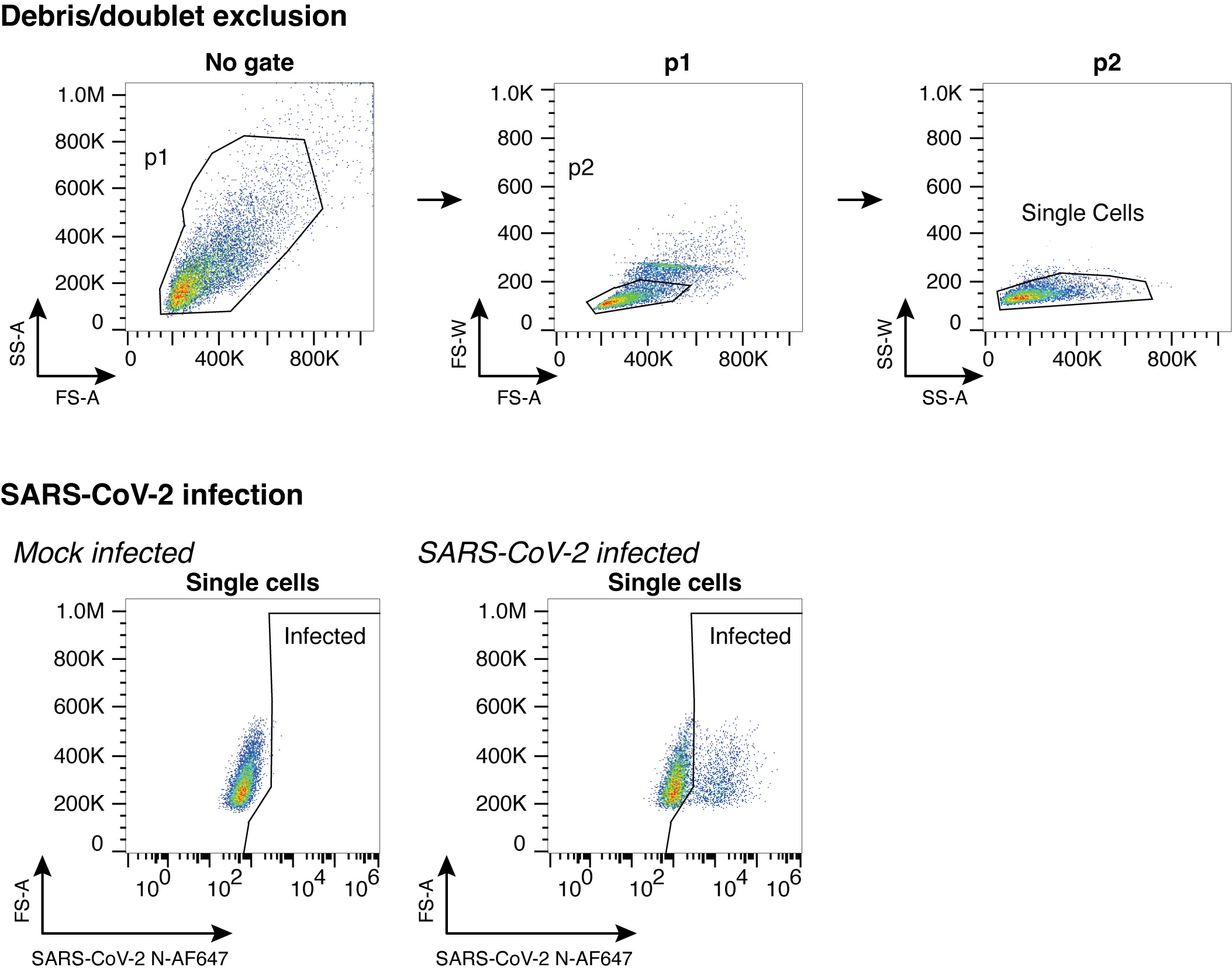

Supplement: S6 Fig — Representative gating strategy for identifying SARS-CoV-2 infected cells. (TIF) [file ppat.1010464.s006.tif]
